# Synchronous delivery of oxygen and photosensitizer for alleviation of hypoxia tumor microenvironment and dramatically enhanced photodynamic therapy

**DOI:** 10.1080/10717544.2018.1435751

**Published:** 2018-02-20

**Authors:** Xiaomeng Guo, Jiaxin Qu, Chunqi Zhu, Wei Li, Lihua Luo, Jie Yang, Xiaoyi Yin, Qingpo Li, Yongzhong Du, Dawei Chen, Yunqing Qiu, Yan Lou, Jian You

**Affiliations:** a College of Pharmaceutical Sciences, Zhejiang University, Hangzhou, Zhejiang, P. R. China;; b Department of Pharmaceutics, School of Pharmaceutical Science, Shenyang Pharmaceutical University, Shenyang, Liaoning, P. R. China;; c State Key Laboratory for Diagnosis and Treatment of Infectious Diseases, Collaborative Innovation Center for Diagnosis and Treatment of Infectious Diseases, The First Affiliated Hospital, Zhejiang University, Hangzhou, Zhejiang, P. R. China

**Keywords:** Tumor hypoxia, photodynamic therapy, oxygen, hemoglobin, photosensitizer

## Abstract

Photosensitizer, proper laser irradiation, and oxygen are essential components for effective photodynamic therapy (PDT) in clinical cancer therapy. However, native hypoxic tumoral microenvironment is a major barrier hindering photodynamic reactions *in vivo*. Thus, we have prepared biocompatible liposomes by loading complexes of oxygen-carrier (hemoglobin, Hb) and photosensitizer (indocyanine green, ICG) for enhanced PDT against hypoxic tumor. Ideal oxygen donor Hb, which is an oxygen-carried protein in red blood cells, makes such liposome which provide stable oxygen supply. ICG, as a photosensitizer, could transfer energy from lasers to oxygen to generate cytotoxic reactive oxygen species (ROS) for treatment. The liposomes loading ICG and Hb (LIH) exhibited efficient tumor homing upon intravenous injection. As revealed by *T*_2_-weighted magnetic resonance imaging and immunohistochemical analysis, the intratumoral hypoxia was greatly alleviated, and the level of hypoxia inducible factor-1α (HIF-1α) and vascular endothelial growth factor (VEGF) in tumor was obviously down-regulated. A weak PDT efficiency was found in cells incubated in simulated hypoxia condition *in vitro*, while PDT effect was dramatically enhanced in LIH treated hypoxia cells under near-infrared (NIR) laser, which was mainly attributed to massive generation of ROS with sufficient oxygen supply. ROS trigger oxidative damage of tumors and induce complete suppression of tumor growth and 100% survival rate of mice, which were also in good health condition. Our work highlights a liposome-based nanomedicine that could effectively deliver oxygen to tumor and alleviate tumor hypoxia state, inducing greatly improved efficacy compared to conventional cancer PDT and demonstrates the promise of modulating unfavorable tumor microenvironment with nanotechnology to overcome limitations of cancer therapies.

## Introduction

Photodynamic therapy (PDT) has been developing as a principal treatment for several types of solid cancer for its superiority of high selectivity, noninvasive, and lower systemic toxicity (Moghissi et al., 2004). It was reported that after PDT treatments, significant growth inhibition was observed in tumor cells, specifically during the G0–G1 phase, by inhibiting carcinogenic mitosis at stages prior to DNA replication (Ahmad et al., 1998). It uses nontoxic photosensitizers and harmless near-infrared (NIR) light to realize the transfer of energy from the oxygen-activated photosensitizer to oxygen, which result in formation of cytotoxic reactive oxygen species (ROS) which cause damage to proteins, lipids, and nucleic acid of cancer cells (Hockel et al., 1996). Thus, photosensitizer, laser irradiation of proper wavelength, and oxygen are all indispensable to realize the therapeutic efficacy of PDT.

It is commonly believed that hypoxia is a characteristic property of solid tumor (Castano et al., 2006) and tumor hypoxic environment have been widely considered to be the main barrier saving the tumor from various therapies (Leone et al., 2015). The oxygen level of solid tumors is fairly low, which contributes to the balance between the supply and the consumption of oxygen in tumor that is destroyed by disturbed microcirculation and deteriorated diffusion (Hockel & Vaupel, 2001). It was reported that the median oxygen partial pressure (pO_2_) values for some normal tissues were from 25–66 mm Hg. While for some kinds of tumors, the value of pO_2_ was even lower than 2.5 mm Hg (Vaupel et al., 1989). Considering the hypoxic microenvironment of tumor, oxygen become the most primary factor which shield cancer cells from the effects of photodynamic therapy. The situation worsened upon light irradiation, the photosensitizer converts oxygen into cytotoxic ROS which aggravate the hypoxia of tumor (Voon et al., 2014). Moreover, vascular shut down due to PDT would also result in severe hypoxia (Busch et al., 2010). The deterioration of tumor hypoxia will promote cancer progression, metastasis, and increase the risk of the resistance of photodynamic therapy (Kimáková et al., 2017). Many methods have been attempted to enhance the efficacy of PDT by increasing the oxygen in tumor or making use of tumor hypoxia state with a bio-reductive pro-drug t irapazamine (TPZ; Liu et al., 2015, 2017). Hyperbaric oxygen inhalation has been used firstly (Jirsa et al., 1991; Maier et al., 2000a), but the vascular damage caused by PDT still prevents further oxygenation from hyperbaric blood (Fingar et al., 1988). The other strategies to enhance reactive oxygen levels in tumor for higher PDT effect were also proposed, which included catalyzing intracellular hydrogen peroxide to oxygen, or improving the solubility of oxygen using perfluorocarbon nanoparticles (Usacheva et al., 2014; Chen et al., 2015; Cheng et al., 2015). However, these strategies were not suitable for enhancing the efficacy of PDT due to the limited oxygen level in tumor under the hypoxic environment or high systemic toxicity. To alleviate hypoxia in tumor cells on the premise of high biosafety may be a key to remarkably improve the efficacy of PDT. Hemoglobin (Hb) is an iron-rich protein in red blood cells, which could deliver oxygen molecules to tissues as the structural basis of Hb could reversibly bind four oxygen molecules to form HbO_2_ (Fleming et al., 2015). Thus, Hb-based oxygen carriers (HBOCs), as a red blood cell (RBC) substitute for blood transfusion, has been widely investigated (Baldwin, 1975; Sakai et al., 2009). In the last few decades, HBOCs of various kinds, such as glutaraldehyde-polymerized Hb and poly(ethyleneglycol)-conjugated Hb (PEG-Hb), have been developed (Alexis et al., 2008; Davis et al., 2008; Lindsay et al., 2016). However, a main side-effect of them is vasoconstriction due to its nitric oxide (NO) scavenging ability, which elicits an acute increase in blood pressure (Yu et al., 2008). Correspondingly, liposome encapsulated Hb is being investigated as a universal oxygen-carrier of nanometric dimensions with decreased side-effects and enhanced circulation half-life for Hb. However, further investigation for their formulation and clinic applications presented a slow progress in recent several years.

**Figure 1.**
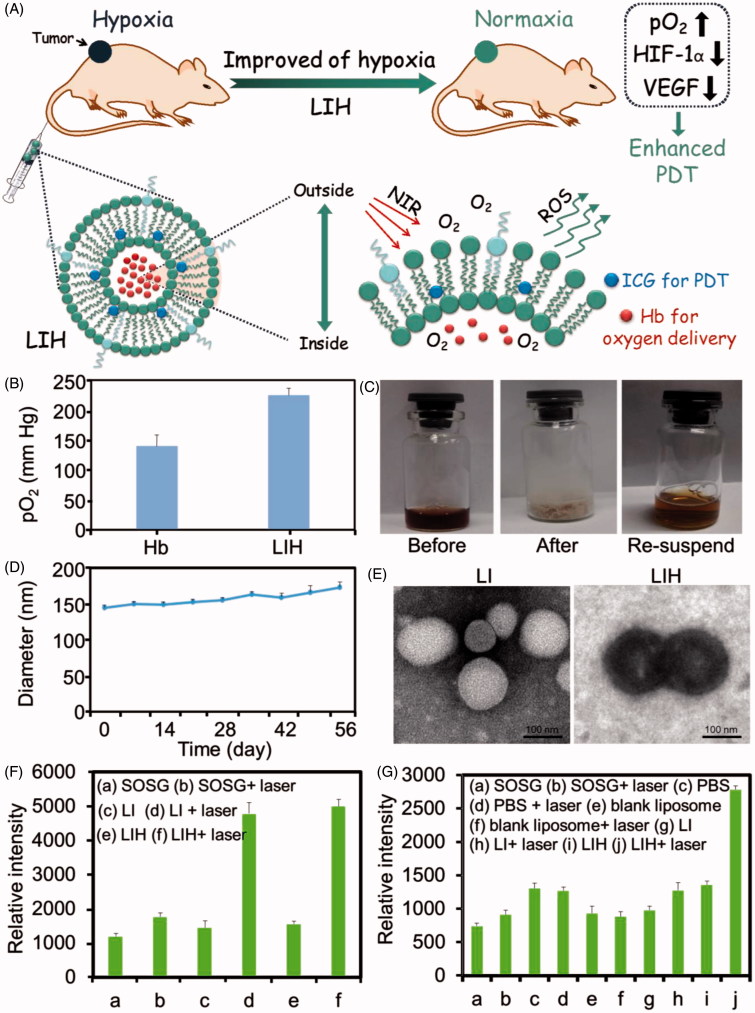
Characterization and ROS generation of LIH. (A) Schematic illustration of alleviation of tumor hypoxia and enhanced PDT based on photosensitizer and hemoglobin co-loaded liposomes (LIH). (B) The pO_2_ of oxygen saturated Hb and LIH at 24 h determined by oximeter. (C) The photos for the LIH solution (before lyophilization), the powder of LIH (after lyophilization), and the LIH solution (the re-suspended). (D) The diameter of LIH in eight weeks after preparation and the concentration of Hb was found to be 2 mg/ml. (E) TEM images of LI and LIH. Scale bar, 100 nm. (F) ROS generation of LI or LIH exposure to NIR laser in normaxia conditions. (G) ROS generation of LI or LIH exposure to NIR laser in hypoxia conditions.

To address the problem of tumor hypoxia and enhance the efficacy of PDT, we designed a oxygen-delivering photosensitive liposome. Hydrophobic indocyanine green (ICG), as a NIR photosensitizer, was modified with octadecylamine (ODA) to increase its hydrophobicity by conjugating the sulfonic group of ICG and the amino group of ODA. Then, ODA modified ICG (ICG-ODA) and Hb loaded liposomes (Lipo-ICG-Hb, LIH) were prepared by loading them into lipid membrane and inner water phase of liposomes, respectively. We hypothesized that the LIH can efficiently penetrate deeply in tumor tissues and synchronously deliver oxygen and photosensitizer into hypoxic tumor. The alleviation of tumor hypoxia was investigated after intravenous injection of LIH into mice bearing tumors and the tumor oxygen level was monitored by *T*_2_-weighted magnetic resonance imaging (MRI). We also work on relieving the hypoxic of tumor by down-regulating the expression of hypoxia inducible factor-1α (HIF-1α) and its downstream vascular endothelial growth factor (VEGF), which was generally believed to be highly expressed in hypoxia (Bergeron et al., 1999; Yee et al., 2009; Long et al., 2012). Upon NIR laser irradiation, ICG accumulated in the tumor could effectively convert nontoxic oxygen to cytotoxic ROS under efficient oxygen supplement. The sufficient oxygen provided by LIH would not only improve the hypoxia of tumor but also enhance the efficacy of PDT. The overall design for LIH and their application is described in Figure 1(A). Taken together, this study provides a strategy for clinical application of nanomedicine in PDT.

## Methods

### Reagents

Human hemoglobin, 1-ethyl-3-(3-dimethylaminopropyl)carbodiimide (EDC), 3-(4,5-dimethy lthiazol-2-yl)-2,5-diphenyltetrazolium bromide (MTT) reagent, and hoechst 33342 were purchased from Sigma (St Louis, MO). 4-Dimethylaminopyridine (DMAP) and ODA were acquired from Aladdin Inc. (Shanghai, China). Singlet oxygen green reagent (SOSG) was acquired from Invitrogen Corp. (Carlsbad, CA). ICG was supplied by TCI (Tokyo, Japan). Distearoyl-sn-glycero-3-phosphoethanolamine-N-[maleimide(polyethylene glycol)-2000] (DSPE-PEG2000), egg phosphatidyl lipid-80 (E80), and cholesterol were purchased from Lipoid GmbH (Ludwigshafen, Germany). ROS detection kit (dichloro-dihydro-fluorescein diacetate (DCFH-DA)) and bicinchoninic acid assay (BCA) protein assay kit were purchased from Beyotime Company (Jiangsu, China). HIF-1α antibody and VEGF antibody were from Proteintech Group, Inc. (Wuhan, China). β-actin antibody and horseradish peroxidase-conjugated secondary antibody were from Santa Cruz Co. Ltd. (Santa Cruz, CA). The hypoxia marker Pimonidazolehydrochloride (Hypoxyprobe-1 plus kit) was purchased from Hypoxyprobe (Burlington, MA). Roswell Park Memorial Institute (RPMI)-1640 medium, fetal bovine serum (FBS), and penicillin/streptomycin (100 U/mL) were from Ji Nuo Biotechnology Co., Ltd. (Zhejiang, China). All other chemicals were of analytical grade and were used without further purification.

### Cell lines and animals

The colon carcinoma cells (CT-26), luciferase-expressing mouse colon adenocarcinoma (CT-26-Luc) cells, and mouse sarcoma (S180) cell lines were purchased from Chinese Academy of Sciences (Shanghai, China). The cell lines were cultured in RPMI 1640 medium containing 10% fetal bovine serum, 1% penicillin and 1% streptomycin at 37 °C in an environment containing 5% carbon-di-oxide (CO_2_). All animal experiments were performed in accordance with the regulations of the Institutional Animal Care and Use Committee (IACUC) of Zhejiang University. BALB/c male mice (18–22 g) and ICR male mice (1 8 ∼ 18–22 g) were raised under aseptic condition in animal isolators with free access to food and water and with an accepted circulation of 12 h light/dark cycles, respectively. During the study, animals were observed for any clinically relevant abnormalities daily. If any animal was moribund due to treatment-associated toxicity, tumor over-growth (∼ 3000 mm^3^), loss of 20% of body weight relative to the start of the study, the appearance of large, or open ulceration was observed before scheduled killing. The rats were sacrificed by CO_2_ inhalation.

### The synthesis of ICG-ODA

The ICG-ODA was synthesized by conjugating the sulfonic group of ICG and the amino group of ODA in the presence of DMAP and EDC. Briefly, ICG, EDC, and DMAP (mass ratio 4:1:1) were dissolved in dimethylformamide and the reaction was allowed to continue for 1 h at 45 °C. After that, the ODA (in water, the mass ratio of ICG:ODA was 2:5) was added and the solution was continued to stirring for another 24 h. The solution was dialyzed against deionized water for 48 h using a membrane (MWCO = 3.5 k Da) and were lyophilized to obtain ICG-ODA.

### Preparation and characterization of LIH

E80 and cholesterol with various molar ratios (10:1, 5:1, 2:1, and 1:1), as a total lipid (10 mg), were dissolved in chloroform. DSPE-PEG_2000_ and ICG-ODA were then added into the above solution (total lipid: DSPE-PEG_2000_: ICG-ODA = 15:1:0.4, molar ratio), and the mixture was warmed to 40 °C in a round-bottomed flask. The solvent in the mixture was evaporated under vacuum in a rotary evaporator until a thin lipid film was formed. one milliliter of PBS solution (pH 7.4) or PBS solution (pH 7.4) containing Hb (2.0 mg/mL) was added into the resulting lipid thin-film with a water bath at 38 °C, followed by stirring. Lipo-ICG (LI) or (LIH was obtained by extruding through filter membranes with 0.8, 0.45, and 0.22 μm pore size in sequence. The average size of LI and LIH was measured by a light scattering method using a Malvern Zetasizer (Malvern, UK) at 25 ± 1 °C. LIH was further lyophilized for their storage. The ability of re-suspension of lyophilized powder to obtain the solution containing LIH was investigated by comparing the size distribution of LIH before and after lyophilization. The UV-visible spectrum of LIH was measured using a spectrophotometer (Agilent Cary 60 UV-Vis, Santa Clara, CA) in the wavelength range of 200–800 nm. The morphology of LIH was investigated using transmission electron microscopy (TEM; JEOL JEM-1230 microscopes, JEOL, Japan). Samples were dried on copper grid and negatively stained with phosphotungstic acid (1%).

Before further experiments, the solution containing LIH was saturated with O_2_ for 15 min for oxygen loading and the oxygen carrying capacity of LIH was detected. Briefly, LIH was dissolved in PBS solution (5 mg/mL) and were saturated with oxygen, then the solution was transferred into a 5 mL bottle filled with argon. The oxygen carrying capacity of LIH was detected by GEM Premier 3000 analyzer (Instrumentation Laboratory Company, Bedford, MA).

### Detection of singlet oxygen (^1^O_2_) *in vitro*

To detect the generation of ^1^O_2_ of LI or LIH, they werre exposed to NIR laser. PBS, LI (8.0 µg ICG/mL) or LIH (8.0 µg ICG/mL) were mixed with SOSG (1.0 μM, dissolved in distilled water containing 1% methanol) in advance. The SOSG fluoroescence of each sample was tested using a fluorescence spectroscopy (Perkin Elmer LS55, Waltham, MA: Excitation and emission wavelength ar 498 and 529 nm, respectively) both before and after the irradiation with NIR laser (808 nm, 1 W/cm^2^, and 1 min).

For the detection of ^1^O_2_ produced by PDT in hypoxia condition (0.05–0.1% O_2_ concentration), the deoxygenated and sealed vials which containing deoxygenated PBS (pH 7.4) were prepared as reaction cells. SOSG (dissolved in distilled water containing 1% methanol) was added at a concentration of 1.0 μM into the reaction cells. Then, PBS, blank liposome, LI (8.0 µg ICG/mL) or LIH (8.0 µg ICG/mL) with the same volume was added into the reaction solution, respectively. Later the fluroescence of each sample was tested both before and after the irradiation with NIR laser (808 nm, 1 W/cm^2^, and 1 min). The sample only containing SOSG without NIR laser irradiation was set as control.

### Cellular uptake

In order to investigate the internalization of LIH into tumor cells, CT-26 colon carcinoma cells were transferred on the glass coverslips in a 24-well plate at a density of 5 × 10^4^ per well and cultured overnight at 37 °C with 5% CO_2_ in air. The medium was then removed and replaced by fresh culture medium containing LIH (25 µg ICG/mL) and the cells were incubated for 1, 2, 4, 6, or 12 h, respectively. After the incubation, the cell nuclei were stained with Hoechst 33342 for 15 min. The cells on the coverslip was repeatedly rinsed with PBS and were mounted for microscopic examination. The ICG fluorescence in cells was examined by a confocal microscope (Olympus, Japan).

The cellular uptake of LI and LIH under normaxia and hypoxia conditions was also determined. CT-26 cells suspended in a medium into six-well plates at a concentration of 2 × 10^5^ cells/well and were allowed to grow overnight. Then, the cells were treated with LI or LIH (25 µg ICG/mL) for 24 h. The plated seeded cells in the hypoxia group were put into an anaerobic bag and the plates in the normaxia group were cultured as usual. The cells were subsequently washed twice with PBS, detached with trypsin- ethylenediaminetetraacetic acid and were resuspended in an appropriate volume of PBS for flow cytometry analysis (FC500 MCL, Beckman Coulter, Brea, CA) and a minimum of 1 × 10^4^ cells were analyzed for each sample.

### Evaluation of hypoxia in cells

The expression of HIF-1α was detected by western blotting to investigate the hypoxia level of tumor cells. CT-26 cells were cultured in normoxia or hypoxia condition for 24 h for adherence. The cells cultured in hypoxia condition were divided into two groups, each group were treated with LIH or PBS for another 24 h, respectively. The cells in normoxia condition were treated with PBS.

For western blotting assay, proteins were extracted from CT-26 cells by radioimmunoprecipitation (RIPA) lysis buffer (Santa Cruz Biotechnology, Dallas, TX). Equal amount of total protein (10 µg) was electrophoresed in a sodium dodecyl sulfate polyacrylamide gel electrophoresis 4–12% Bis-Tris Gel (Life Technologies, Carlsbad, CA) and were transferred to nitrocellulose membranes (Shanghai Bioscience Biotechnology Co., Ltd. Shanghai, China). The membrane was blocked with 10% nonfat dry milk in tris-buffered saline and 0.1% Tween 20 (TBST) for 2 h at room temperature, followed by incubating with anti-HIF-1α and β-actin antibody overnight at 4 °C. After washing with TBST, the membrane was incubated with horseradish peroxidase-conjugated secondary antibody at room temperature for 4 h. Labeled proteins were detected using chemical luminescence (ECL Advanced Western Blotting Detection Kit, GE Healthcare Life Sciences, Marlborough, MA).

For the immunofluorescence staining of HIF-1α, the cells were fixed with 4% formaldehyde and were labeled with anti-HIF-1α antibody for 12 h. Then, they were incubated with Cy3-labeled Goat anti-mouse immunoglobulin G for 4 h. Finally, the nucleus was stained with Hoechst 33342. The fluorescent signals were observed by a confocal laser scanning microscope (CLSM, Carl Zeiss, Jena, Germany).

### ROS detection *in vitro*

The ROS production of LI or LIH exposed to NIR laser in cells under normoxia or hypoxia condition was studied using DCFH-DA. Firstly, CT-26 cells were planted and allowed to adhere for 24 h at 37 °C in normoxia or hypoxia environment. The cells were incubated with LI (60 μg ICG/mL) or LIH (60 μg ICG/mL and 200 μg Hb/mL) for another 4 h and were also treated with DCFH-DA (10 μM) at 37 °C for 20 min. Later the cells were irradiated by NIR laser (1 W/cm^2^and 1 min). After 30 min, the fluorescence and bright field images of the cells were measured by fluorescence inversion microscope system (F900, Edinburgh Instruments Ltd., UK) with emission at 522 nm and excitation at 488 nm.

### Cytotoxicity

CT-26 cells were seeded into 96-well plate at a density of 5 × 10^3^/well and were incubated overnight at 37 °C in normoxia (20% oxygen concentration) or hypoxia environment (0.05–0.1% oxygen concentration). Then, the medium was removed and replaced with 200 µL of fresh culture medium containing different concentration of LI or LIH. To simulate hypoxia environment, the 96-well plates with cells were put into an anaerobic bag with an oxygen indicator (MGC AnaeroPack^TM^ Series, Mitsubishi gas chemical, Japan). As the oxygen was consumed by AnaeroPack, the oxygen indicator changed its color from blue to purple or pink, which demonstrated the oxygen concentration in hermetic bag was less than 0.1%. The hypoxia experiments were performed under the protection of argon. For the NIR laser group, the cells were incubated with LI or LIH for 4 h, followed by an irradiation of NIR laser (808 nm, 1 W/cm^2^, and 1 min). After further incubation of 48 h, MTT method was used to test the cytotoxicity. The data were expressed as the percentage of surviving cells and are reported as the mean values of three measurements. The Annexin V- fluorescein isothiocyanate (FITC)/ propidium iodide double staining was also used to investigated the enhanced PDT of LIH *in vitro*.

### Biodistribution

Subcutaneous S180 and CT-26 tumor models were obtained by subcutaneously injecting a suspension of S180 cells (∼1 × 10^6^ cells) and CT-26 (∼1 × 10^6^ cells) into ICR and BALB/c mice, respectively. The experiments were carried out when the tumor volume was about 200 mm^3^. Orthotopic tumor models are considered more clinically relevant and better predictive models of drug efficacy than standard subcutaneous models. In this work, deep orthotopic CT-26 tumor model was also established by directly injecting CT-26-luc cells (∼1 × 10^6^ cells) into the colorectal membrane of BALB/c mice. Four to eight days later, the tumor growth was monitored by observing the bioluminescence signal using the IVIS Spectrum Imaging System (caliper, Perkin Elmer) after injection of luciferin.

For *in vivo* real-time optical imaging, the mice bearing tumor were intravenous injected with LIH (0.6 mg ICG/mL and 2 mg Hb/mL) or LI (0.6 mg ICG/mL). Fluorescence images of tumors were taken at 2, 6, 12, 24, 36, and 48 h, respectively after injection using the *ex*/*in vivo* imaging system (CRI Maestro, Woburn, MA), with a 704 nm excitation wavelength and a 735 nm filter to collect the fluorescence signals of ICG. Fluorescent images of LIH in the orthotopic CT-26 tumors were acquired at 24 h post injection by Maestro imaging system (for the determination of ICG fluorescence) and IVIS Spectrum Imaging System (for the determination of bioluminomescence from CT-26 tumor cells). The quantitative data were analyzed by Image J software (NIH, Bethesda, MA). The mice were sacrificed at the end of experiments and the organs including heart, liver, spleen, lung, kidney, and tumor were collected for fluorescent imaging and quantitative analysis.

### Evaluation of hypoxia *in vivo*

To evaluate the hypoxia alleviating ability of LIH *in vivo*, both xenograft and orthotopic CT-26 tumor model were used. The tumor bearing mice were randomly divided into three groups and were intravenously injected with saline, LI (100 μL, 6.0 mg total lipid per mL), and LIH (100 μL, 6.0 mg total lipid per mL and 10 mg Hb per mL) for three times (one injection per day), respectively. The tumors were dissected at 24 h after the last injection and were cut into 5 μm slices. For the detection of hypoxia in tumors, the tumor slides were stained using a commercial Hypoxyprobe-1 plus kit (Hypoxyprobe, Burlington, MA) following the protocol provided. For the detection of HIF-1α and VEGF, the tumor slides were incubated with polyclonal rabbit anti-HIF-1α antibody (dilution1:100) and VEGF antibody (dilution 1:100), respectively. After washing with PBS, the HIF-1α, and VEGF antibody was detected using FITC conjugated goat anti-rabbit immunoglobulin G, followed by observation using a confocal microscope.

To further investigate how long the alleviated tumor hypoxia state could maintain, the tumors were dissected at day 2, 4, and 7 after the last injection of LIH. The expression of HIF-1α and VEGF in tumor was determined using the methods as the mentioned above and the degree of hypoxia in tumor also was analyzed using Hypoxyprobe-1 plus kit.

### MRI imaging for the evaluation of hypoxia *in vivo*

To evaluate tumor hypoxia directly, *T_2_*-weighted MRI was also used. The mice bearing tumor were intravenous injected with saline, LI (0.6 mg ICG/mL), or LIH (0.6 mg ICG/mL and 2 mg Hb/mL), respectively. MRI images of subcutaneous tumors were taken pre and post two days after injection using the GE discovery 3.0 T clinical MRI scanner (GE discovery 740, GE Healthcare) equipped with a horizontal bore. For tumor imaging, *T_2_*-weighted images were obtained using a spin echo multiple slice sequence with a repetition time (TR) of 2500 ms and an effective echo time (TE) of 33 ms. The *T_2_* relaxation time was measured using a multislice multiecho technique with a TR of 2500 ms and 16 TE of 11–176 ms. The *T*_2_* relaxation time was measured using a multigradient echo technique with a TR of 1500 ms, 12 TE values of 3.8–45 ms, and a flip angle of 30°.

### ROS detection *in vivo*

The mice bearing subcutaneous CT-26 tumors were divided into three groups when the tumor size was over than 150 mm^3^ (*n* = 3). The mice in group 1–3 were intravenously injected with saline (three injections with one injection per day), LI (100 μL, 6 mg total lipid per mL, three injections with one injection per day), and LIH (100 μL, 6 mg total lipid per mL and 10 mg Hb per mL, three injections with one injection per day), respectively. At 24 h after the last injection, all mice were sacrificed. The tumors were dissected and cut into 5 μm slices for the detection of ROS. The slices of each group were incubated with DCFH-DA (10 μM) at 37 °C for 20 min and then irradiated by NIR laser (1 W/cm^2^and1 min), respectively. After 30 min, the fluorescence and bright field images of the cells were measured by fluorescence inversion microscope system with emission at 522 and excitation at 488 nm, respectively.

### PDT effect *in vivo*

Subcutaneous CT-26 xenograft tumor models were selected to evaluate the efficacy of PDT with a combination of the alleviation of tumor hypoxia via the mediation of LIH. The mice bearing tumor were randomly divided into four groups labeled as saline, LI plus laser, LIH, and LIH plus laser. The mice in group 1–4 were intravenously injected with 100 μL of saline, LI (3.2 mg ICG/mL), and LIH (3.2 mg ICG/mL and 3.2 mg Hb/mL) or LIH (3.2 mg ICG/mL and 3.2 mg Hb/mL), respectively for three times (every two days). For laser treatment groups, the tumors were irradiated by NIR laser (808 nm, 1.5 W/cm^2^, and 3 min) at 6 and 24 h after each injection. Tumor volume was recorded using a caliper and the tumor volume was determined as (length × width^2^)/2. At the end of the experiments, the heart, liver, spleen, lung, kidney, and tumors in each group were dissected to make paraffin section for hematoxylin and eosin (H&E) staining. The proliferation and apoptosis of tumors in each group were also detected by Antigen KI-67(Ki67) and terminal deoxynucleotidyl transferase (TdT) dUTP Nick-End Labeling (TUNEL) staining. The slices were observed by an inverted phase contrast microscope (Nikon, Japan).

### Statistical analysis

The results are presented as the mean ± SD. Statistical comparisons were conducted using paired t-tests with a two-tailed *p* value to compare selected data pairs when only two groups were compared. If more than two groups were compared, an evaluation of significance was performed using a one-way analysis of variance (ANOVA). All statistical analyses were conducted using GraphPad Prism software (GraphPad software, La Jolla, CA).

## Results

### Characterization

E80, hydrogenated soybean phosphatidylcholine (HSPC), Lipoid S100, and DPPC were chosen to prepare LIH liposomes and the stability of prepared liposomes were evaluated by UV-Vis spectrum. Finally, E80 was selected for its good biocompatibility and lower phase-transition temperature (−8 °C). Various molar ratio of E80 to cholesterol (10:1, 5:1, 2:1, and 1:1) were investigated. Although there was no significant difference in the appearance of LIH prepared with different mole ratio of E80 to cholesterol (Supplementary Figure S1), 2:1 were more suitable for the stability of the liposomes. The encapsulation efficiency and content of Hb in LIH was 62.3 ± 4.3%, determined by the UV-Vis spectrum at the wavelength of 415 nm. Furthermore, we investigated the stability of oxygen-carrying LIH (Figure 1(B)). The pO_2_ of oxygen saturated Hb and oxygen saturated LIH at 24 h were 140 and 230 mm Hg, respectively, which showed that the ability of combining oxygen of LIH was better than free Hb. Oxygen molecules in LIH release slower and LIH was more suitable for further use. This is probably due to the phospholipid bilayer membrane, which protects the oxygen saturated Hb and slows down the rate of oxygen release.

The obtained LIH could be lyophilized for long term storage. It was found that the re-suspension of LIH was easily obtained by putting the powder into pure water. Figure 1(C) showed that the lyophilized LIH powder could be stored at room temperature for two months and then was re-suspended to obtain the solution containing LIH without significant changes of appearance compared to that of the original LIH. Figure 1(D) showed the diameter of re-suspension of LIH after lyophilized LIH powder was stored at room temperature for eight weeks, indicating the tiny changes of LIH diameter. The morphology of LI and LIH presented a spherical shape, observed by TEM. The ferrous (Fe^2+^) in hemoglobin encapsulated in liposomes induced a deep color for LIH (Figure 1(E)).

### *In vitro*^1^O_2_ detection

The impact of laser irradiation and different oxygen level conditions (normoxia: 21% O_2_ concentration or hypoxia: 0.05–0.1% O_2_ concentration) on the ^1^O_2_ generation of liposome solutions were investigated. In normaxia condition, it is obviously observed that LI and LIH solution both generated ^1^O_2_ with the irradiation of NIR laser (Figure 1(F)). In the conditions without laser, there were no significant difference between the fluorescence of the LI group, LIH group, and control group (SOSG). This indicates that the photosensitizer and proper NIR light are necessary for photosensitive liposomes to produce ^1^O_2_.

To further investigate the influence of oxygen level, we simulated hypoxia environment with the reaction vials (deoxygenated and sealed vials). The oxygen saturated PBS, blank liposome, LI, and LIH were injected into reaction vials, respectively and the fluorescence intensity of each group was detected before and after laser irradiation. From Figure 1(G) we can see that the fluorescence intensity of PBS and blank liposome groups increase which was not so obvious, as there is no photosensitizer to convert the oxygen into ^1^O_2_. However, the fluorescence intensity of LI group also exhibited unobvious increase, even if the photosensitizer exists. The interest thing is that the reaction vial injected with LIH showed about two-fold in generation amount of ^1^O_2_ than other groups. Although the generation of ^1^O_2_ in hypoxia condition was less than in normaxia condition, it still proved that LIH could carry oxygen and provide oxygen molecules for ICG to convert oxygen to ^1^O_2_ in hypoxia condition.

### Cellular uptake

**Figure 2.**
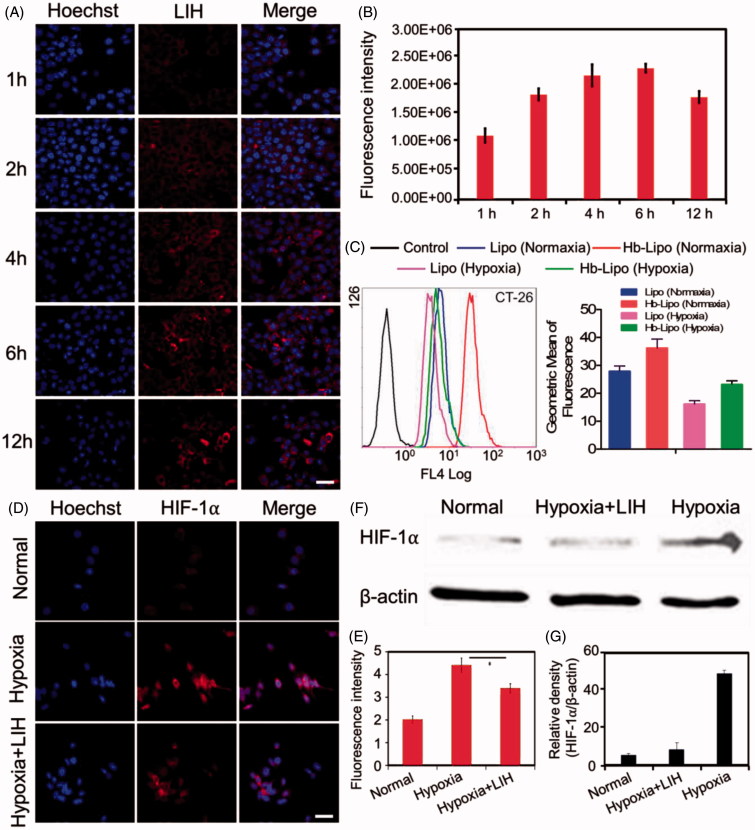
Cellular uptake and hypoxia alleviation of LIH in CT-26 cells. (A) Confocal microscopy images of cellular uptake of LIH liposomes in normaxia condition. Scale bar, 50 μm. (B) The semi-quantitative analysis of fluorescence intensity in (A) determined by ‘Image J’ software. (C) Cellular uptake of LIH liposomes at 24 h in CT-26 cells determined by a flow-cytometer under normaxia and hypoxia conditions. (D) Immunostaining analysis of HIF-1α expression in CT-26 cells under different incubation environment. Scale bar, 50 μm. (E) The quantitative analysis based on the images in (D) by ‘Image J’ software. (F) Western blot analysis of HIF-1α expression in CT-26 cell lines. (G) Semi-quantitative analysis of percentage in (F). All the data were analyzed by one-way ANOVA (*p ≤ .05; **p ≤ .01).

The CT-26 cellular uptake of LIH is shown in Figure 2(A), which was tested by determining the fluorescence of ICG. The results showed the fluorescence intensity in CT-26 cells increased gradually with the expending of incubation time, which indicated that LIH could efficiently internalize into tumor cells and accumulate in the cytoplasm. Semi-quantitative analysis showed a quick uptake of LIH into the cells in short incubation time (0–4 h) and subsequently a saturation tendency of LIH internalization (Figure 2(B)).

The CT-26 cellular uptake of LI and LIH under normaxia and hypoxia conditions was also determined. From Figure 2(C) we can see that the both LI and LIH were easy to be internalized by cells under normaxia than those in hypoxia conditions. After 24 h of incubation, the average ICG fluorescence intensities in cells with the LI and LIH in normaxia condition was 1.35- and 1.18-fold higher than those incubated under hypoxia condition, respectively. Interestingly, we found that the internalized amount of LIH was higher than LI both under normaxia and hypoxia conditions and the Hb might enhance the cellular uptake of liposomes to some extent. It is reported that the similar RBC nanosystems exhibit excellent biological barriers overcoming abilities. The completeness of membrane coverage is so important, since it can shield the nanoparticles from external exposure, thereby minimizing the risk of association with foreign materials. Thus, this bionic nanoparticle might be more stable and easier to be internalized into cells (Luk et al., 2014; Chen et al., 2017).

### The influences of LIH on hypoxia *in vitro*

Since HIF-1α is a reliable indicator of the degree of tumor hypoxia, the high expression of HIF-1α in cells with hypoxia culturing indicated a successful simulating of tumor hypoxia microenvironment. The results of HIF-1α fluorescence immune staining is shown (Figure 2(D,E)), it can be seen that the high expression level of HIF-1α could be down-regulated by the addition of LIH under the hypoxia condition. From the results of western-blotting (Figure 2(F,G)) we also easily found that the expression of HIF-1α in hypoxia condition was about 10 times higher than that in normaxia condition. However, the application of LIH in hypoxia condition could alleviate hypoxia efficiently. Although LIH didn’t reverse the hypoxia, its down-regulating of the expression of HIF-1α was obviously observed in hypoxia condition. These all confirmed the oxygen carrying ability of LIH and proved that LIH can effectively deliver oxygen into the cells and improve cell hypoxia.

### ROS detection *in vitro*

DCFH-DA itself did not exhibit any fluorescence, but it can pass through the cell membrane freely into the cell and can also be hydrolyzed by intracellular esterase to DCFH. While DCFH can’t penetrate through the cell membrane, thus the probe can be easily internalized by the cell. Intracellular ROS can oxidize non-fluorescent DCFH to produce fluorescent DCF. Fluorescence of DCF could be detected to determine the level of intracellular ROS.

**Figure 3.**
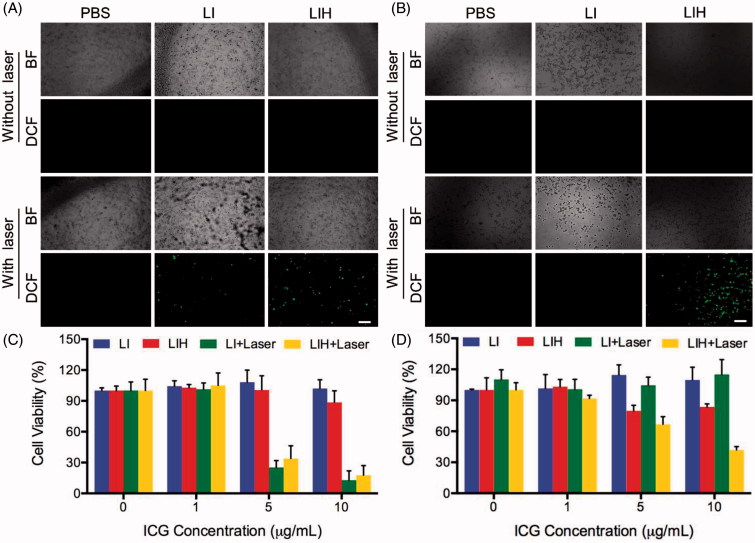
PDT efficiency of LIH. Intracellular ROS detection of PBS, LI, and LIH under (A) normaxia and (B) hypoxia condition with or without laser irradiation (808 nm, 1 W/cm^2^, and 1 min). Cytotoxicity of LI and LIH against CT-26 cells in (C) normaxia and (D) hypoxia environment without or with laser irradiation (808 nm, 1 W/cm^2^, and 1 min).

In normoxia condition, the cells treated with PBS, LI, and LIH didn’t generate any ROS without NIR laser irradiation, but ROS production were both observed in the cells treated with LI and LIH which was detected by the fluorescence of DCF (Figure 3(A)). Compared to LI, the LIH produced more ROS because it carries more oxygen into the cell by oxygen saturated Hb. While in hypoxia condition, without the irradiation of NIR laser, the cells still couldn’t generate any ROS. This time even the cells treated with LI couldn’t generated ROS, but magically, the generation of ROS in the cells treated with LIH was clearly observed (Figure 3(B)). This is more fully illustrated that the LIH could carry oxygen into the cells and provide oxygen for the photosensitizer to produce ROS for PDT. In order to achieve effective PDT, photosensitizer, laser, and oxygen are indispensable.

### Cell cytotoxicity

After comparing the performance of LI and LIH as ^1^O_2_ producer, we tested their photodynamic effects on inducing cell death. In normoxia condition, there was no significant cytotoxicity in CT-26 cells treated with LIH or LI without NIR irradiation (Figure 3(C)). While after irradiation, the reduction in the viability of cells treated with both LI and LIH were detected (Figure 3(C)). It is notable that the viability in cells treated with LI and LIH were reduced when the concentration of ICG were higher than 5 µg/mL. The effects of PDT in hypoxia were also studied. Interestingly, in hypoxic conditions (Figure 3(D)), PDT mediated by LIH still maintained superior cytotoxicity to PDT mediated by LI (*p* < .05), indicating that LIH can improve the efficacy of PDT in both normal and hypoxia conditions. The results of Annexin V-FITC/PI also demonstrated the enhanced PDT effect of LIH in hypoxia condition (Supplementary Figure S2). This may be attributed to LIH that could provide sufficient oxygen for PDT in hypoxia.

### Biodistribution

Because of the enhanced permeability and retention effect (EPR), nanoparticles in the range of 10–400 nm can accumulate in tumor site at levels 70 times higher than in normal tissues. Accordingly, our LIH with around 200 nm diameter could passively target to tumors.

**Figure 4.**
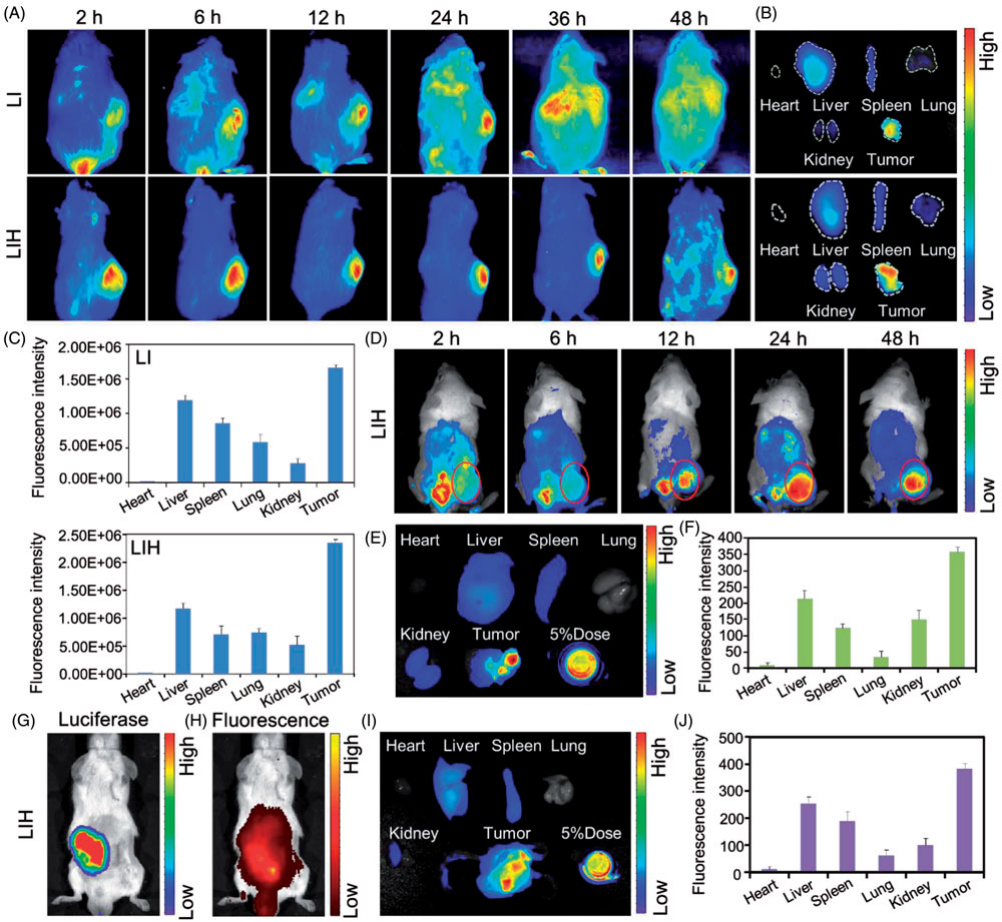
Biodistribution of LIH in xenograft S180 , CT-26 tumor, and orthotopic CT-26 tumor model mice. (A) *In vivo* fluorescence imaging of LI and LIH in S180 tumor bearing ICR mice post intravenously injection. (B) The fluorescent images of various tissues at 24 h after injection of LI and LIH. (C) The LI and LIH in various tissues were calculated as the average fluorescent intensity, representing the amount of the liposomes. (D) *In vivo* fluorescence imaging of LIH in CT-26 tumor bearing ICR mice post intravenously injection. (E) The fluorescent images of various tissues at 48 h after injection of LIH. (F) The LIH in various tissues was calculated as the average fluorescent intensity, representing the amount of the liposomes. (G) In *vivo* bio-luciferase imaging of orthotopic CT-26-Luc tumor after injection of D-luciferin. (H) *In vivo* fluorescence imaging of LIH at 24 h post injection. (I) The fluorescence imaging of organs and tumor of orthotopic CT-26-Luc tumor bearing mice at 24 h after intravenously injection of LIH. (J) The LIH in various tissues was calculated as the average fluorescent intensity.

Obvious fluorescence signals increase of LIH in S180 tumors was presented with the extension of time after injecting LIH into the mice bearing the tumors (Figure 4(A)), indicating the efficient retention of LIH in the tumors for at least 48 h. Figure 4(B,C) showed the fluorescence and semi-quantitative analyze of LI or LIH in main organs and tumors. More LIH accumulated in tumors than other main organs (even liver) and it is noticeable that the amount of LIH was more than that of LI in tumors, which means Hb encapsulated in the liposome could make LIH accumulate in tumors more efficiently. The semi-quantitative analysis also showed a similar result. For the CT-26 subcutaneous tumor bearing mice, the LIH accumulated most in the tumor at 24 h after injection (Figure 4(D)). After 48 h, it can be seen that LIH accumulated most in the tumor (Figure 4(E,F)).

Orthotopic tumor models are considered more clinically relevant and better predictive models of drug efficacy than standard subcutaneous models. In order to investigate the accumulation of LIH in orthotopic tumors, a visual orthotopic colon tumor model was established by injecting CT26-Luc cells with the expression of insect luciferase into the colorectal membrane after an abdominal surgery. It was found that strong bioluminomescence was observed on abdomen under the IVIS Spectrum Imaging System after 4–8 days of the surgery (Figure 4(G)). The fluorescence image indicated the retention of LIH in the tumors at 24 h after injection, which was co-localized with the bioluminomescence from CT-26 cells to a large extent (Figure 4(H)). After the dissection of the mice, it was also observed that strong fluorescence signal of LIH was found in orthotopic CT-26 tumor (Figure 4(I,J)).

All of above proved the effective passive targeting of LIH to tumors strongly.

### The influences of LIH on hypoxia *in vivo*

**Figure 5.**
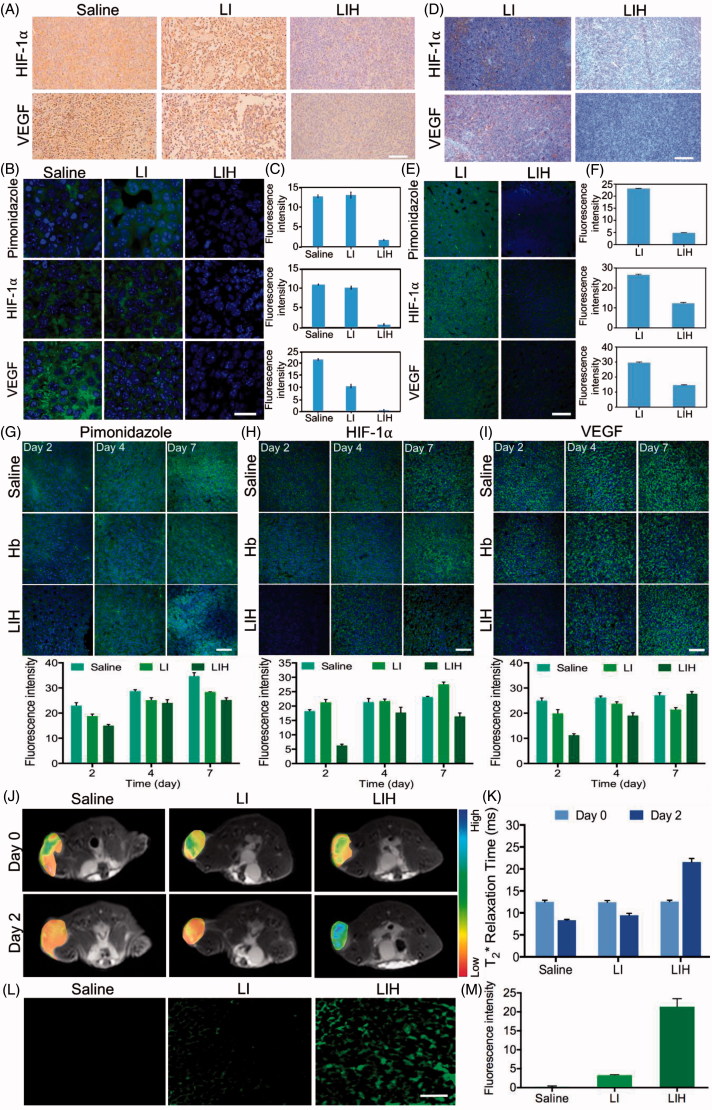
Effects of LIH on tumor hypoxia alleviation. (A) The expression of HIF-1α and VEGF in CT-26 subcutaneous tumor model mice treated with saline, LI, or LIH. Scale bar, 100 μm. (B) Representative immunofluorescence images of tumor slices after hypoxia staining. Scale bar, 50 μm. (C) The relative hypoxia positive area, HIF-1α ,and VEGF receptor were recorded from more than ten micrographs for each group by ‘Image J’ software. (D) The expression of HIF-1α and VEGF in CT-26 orthotopic tumor of mice treated with LI or LIH. Scale bar, 100 μm. (E) Representative immunofluorescence images of tumor slices after hypoxia staining. Scale bar, 100 μm. (F) The relative hypoxia positive area, HIF-1α,and VEGF receptor were recorded from more than 10 micrographs for each group by ‘Image J’ software. (G) Representative immunofluorescence images and semi-quantitative analysis of tumor slices after hypoxia staining by antipimonidazole antibody (green). Scale bar, 100 μm. (H) Representative immunofluorescence images and semi-quantitative analysis of tumor slices after HIF-1α staining by anti-HIF-1α antibody (green). Scale bar, 100 μm. (I) Representative immunofluorescence images and semi-quantitative analysis of tumor slices after VEGF staining. Scale bar, 100 μm. (J) Effect of liposomes on tumor oxygen on day 2 after intravenously injection of LI or LIH. A significant increase in T_2_* values of tumors treated with LIH at day 2 after injection. (K) T_2_-weighted MR image intensity in (J). (L) Representative intratumoral DCFH-DA fluorescence images as an indicator of ROS generation level. Scale bars, 50 μm. (M) Mean fluorescence intensity of DCFH-DA (ROS) inside the tumors.

After injecting saline, LI, and LIH into the mice for three times (one injection per day) respectively, CT-26 tumors were collected for the determination of HIF-1α and VEGF expression by immunohistochemical analysis. In saline and LI groups, the tumor slides showed a deep brown color, demonstrating the high expression of HIF-1α in the tumors. While, only light brown was presented in the tumors of LIH group, indicating low HIF-1α and VEGF expression (Figure 5(A)). The results indicated that the administration of LIH could induce the change of hypoxia into oxygen rich microenvironment in the tumors. As a target gene of HIF-1α, VEGF transcription in hypoxic cells is up-regulated by HIF-1α, a deep brown color which represents high expression of VEGF was also observed in saline group and LI group, only light brown was presented in the tumors of LIH group. Reducing the expression of HIF-1α and VEGF in the tumors mediated by LIH was further confirmed by fluorescence immune staining (Figure 5(B,C)). Pimonidazole as the main component of Hypoxyprobe-1 will be automatically accumulated in the hypoxia site. We investigated the effect of LIH on tumor hypoxia immunofluorescence staining to determine tissue hypoxia using a Hypoxyprobe-1. The tumor showed less tissue hypoxia, decrease in the expression of HIF-1α, and decrease in the expression of VEGF compared to those in saline treatment group (Figure 5(B,C)), indicating the ability of LIH to reduce tumor hypoxia.

After the administration of the same Hb dose, LIH also significantly affect the hypoxia microenvironment in orthotopic CT-26 tumors. Figure 5(D) shows the obvious decreased HIF-1α and VEGF expression in the tumors of LIH group, but high HIF-1α expression in the tumors of LI group, which further demonstrated the ability of LIH to reduce the level of tumor hypoxia. The expression of VEGF further induces angiogenesis and plays a key role in promoting malignant tumor growth. Thus, down-regulation of HIF-1α and VEGF expression by alleviating tumor hypoxia would inhibit tumor progression. Clearly, LIH also induced the significant down-regulation of VEGF expression in the orthotopic colon tumors. Fluorescence immune staining of HIF-1α, VEGF, and hypoxia confirmed the improvement of hypoxia by LIH (Figure 5(E,F)).

HIF-1α produced by tumor cells plays a vital role in an adaptive response to hypoxia by modulating various cellular functions like proliferation, apoptosis, angiogenesis, and anaerobic glycolysis (Denko, 2008). While activated, HIF-1α binds to the hypoxia responsive element, thereby promoting transcription of various genes including VEGF. The expression of VEGF further induces angiogenesis and plays a key role in promoting malignant tumor growth (Carmeliet & Jain, 2000). Thus, down-regulation of HIF-1α and VEGF expression by alleviating tumor hypoxia would inhibit tumor progression.

Figure 5(G-I) showed further investigation of how long the LIH maintain the improvement of tumor hypoxia environment will last. The detection of tumor hypoxia showed LIH could alleviate tumor hypoxia for two days at least, obviously decreased of HIF-1α and VEGF expression still exist two days post the last injection of LIH. But with the extension of time, tumors hypoxia recovered, and an obviously increase of HIF-1α and VEGF was observed. Therefore, LIH could improve the tumor hypoxia and this influence will last for at least two days, which is very important to confirm the administration time point of *in vivo* experiment.

### MRI imaging for the evaluation of hypoxia *in vivo*

The effect of LIH on tumor oxygenation was assessed using blood oxygen level-dependent MRI (Kami et al., 1995; Jiang et al., 2013; Hall et al., 2014; Kim et al., 2014; Zhao et al., 2015). The theory is that the paramagnetic deoxyhemoglobin creates microscopic field gradients, which enhance the transverse relaxation rate, *R*_2_*, of water protons in blood and in the tissue adjacent to blood vessels. Decrease in deoxyhemoglobin concentration leads to a decreased *R*_2_*, and thus, an increased signal intensity in *T*_2_*-weighted MRI. We performed *T*_2_*-weighted MRI on CT-26 tumor mice at before and two days after injection of saline, LI, or LIH and observed an obvious increase in *T*_2_* values of tumors treated with LIH liposomes (Figure 5(J,K)), indicating increased oxygen concentration in the tumor blood. Furthermore, the significant increase in *T*_2_* value of tumor was not only in the peripheral region, but also in the tumor core in which hypoxic region is located. On the contrary, the *T*_2_* values of tumors treated with saline and LI were a little decreased. The advantage of using LIH is the ability to increase oxygen concentration in the tumor area by delivery of LIH which are highly accumulated in tumor and could release oxygen into tumor cells.

### ROS detection *in vivo*

After the xenograft CT-26 tumor bearing mice were treated with saline, LI, or LIH, respectively for three times (one injection per day), the tumors were dissected and exposed to 808 nm laser (1 W/cm^2^and 1 min), after which the generation of ROS was detected by DCFH-DA. The results are shown in Figure 5(L,M), no matter whether the tumor is hypoxia or normaxia, there is no doubt that the ROS couldn’t be generated without photosensitizer, thus it is not surprising that the saline group couldn’t produce any ROS. Although both LI and LIH provided photosensitizer for tumor, the generation of ROS in two groups showed significant difference. The tumor of mouse which treated with LIH apparently produced more ROS than the mouse treated with LI. It demonstrates that although the amount of photosensitizer was same, LIH would provide more oxygen for ICG to produce ROS under the irradiation of NIR laser.

These results give a strong validation that LIH could deliver oxygen molecules to tumors, improve the hypoxia microenvironment of tumors, and provide oxygen for photosensitizer to produce ROS.

### PDT effect *in vivo*

**Figure 6.**
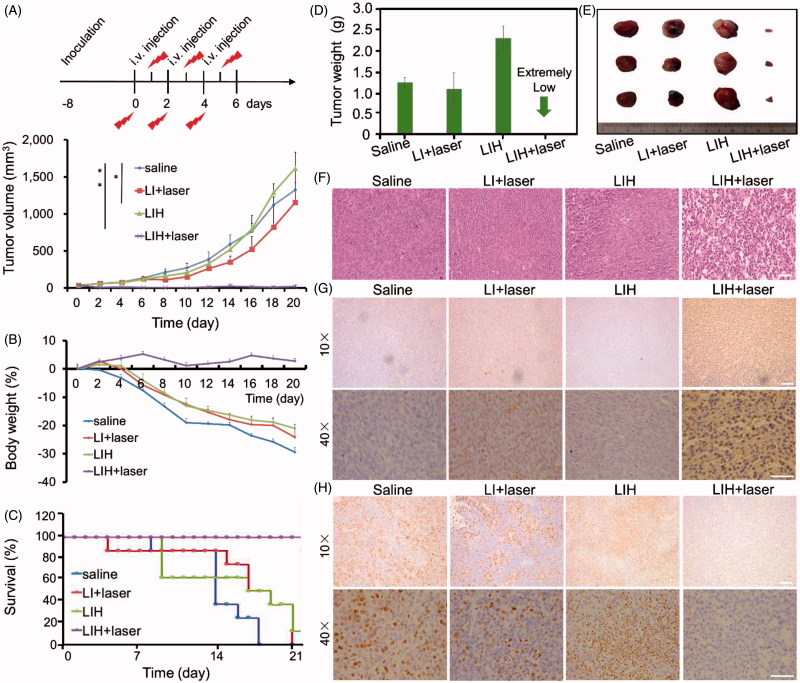
*In vivo* enhanced PDT with LIH. (A) Schematic of the time course of the efficacy study. Tumors growth curves for the mice bearing CT-26 tumors after being treated with saline, LI plus laser, LIH, and LIH plus laser. The tumors were irradiated by NIR laser at 24 h after intravenous injection (808 nm, 1.5 W/cm^2^, and 3 min). (B) Change of mice body weight noted within 19 days . (C) Survival rates of mice within 21 days. (D) Average tumor weight after treatment. (E) Representative tumor image of various groups. (F) Histological observation of tumor tissues via H&E staining. Scale bars, 50 μm. (G) Detection of PDT-induced apoptosis in tumor tissues with TUNEL staining assay. Blue signal: cell nucleus; green signal: apoptotic cells. Scale bars, 50 (10×) and 100 μm (40×). (H) Detection of proliferation in tumor tissues with Ki67 staining assay. Blue signal: cell nucleus; green signal: proliferative cells. Scale bars, 50 (10×) and 100 μm (40×).

To evaluate the PDT effect of LIH *in vivo*, CT-26 xenograft tumor bearing mice were divided into four groups and were treated with saline, LI plus laser, LIH, or LIH plus laser, respectively. After intravenously injected with LI or LIH, the laser groups were followed with twice exposure to NIR laser at 6 and 24 h post injection. The time course of the efficiency study and the CT-26 tumor growth curves of mice are shown in Figure 6(A). After three weeks, the tumor treated with saline, LI plus laser, and LIH grew rapidly and the tumor size were as large as 1326 ± 96.87, 1156 ± 472.66, and 1617 ± 215.15 mm^3^, respectively. However, the treatment with photosensitive liposomes encapsulated with oxygen saturated Hb plus laser (LIH plus laser) showed significantly higher antitumor activity than the other three treatments. Interestingly, the tumor treated with LIH grew faster than saline, which indicated that the photosensitizer couldn’t converts oxygen into cytotoxic ROS to inhibit the growth of tumor without laser, but the LIH provide adequate oxygen to tumor for its malignant growth. While for the group of treated with LI plus laser, the low concentration of oxygen in tumors hinder the effect of PDT. Although the tumor growth rate was slower than the saline group, no significant inhibition of tumor growth was observed. From the picture of tumor captured 2–18 days (Supplementary Figure S3), we can find the same results to match the growth curve of tumor. To sum up, these results demonstrated the importance of photosensitizer, NIR laser, and adequate oxygen, which are all indispensable.

The weight variations were also monitored during the experiments every two days (Figure 6(B)). The body weight of mice treated with LIH plus laser continued to increase for the whole experimental period, resulting in a 2.77% increase at the end of experiment. However, the weight of mice treated with saline kept declining all the time. LI plus laser or LIH groups showed slight weight increase during the first four days, then the weight of mice continued to decline, which reflected the bad condition of mice with non-effective treatments. The body weight of mice in LIH plus laser group was kept at a relative stable state for achieving effective treatment. The survival curves of these four groups are shown in Figure 6(C). Clearly, the life span of mice treated with LIH plus NIR laser irradiation was the longest, with a survival rate of 100% until day 21. In contrast, mice in the saline, LI plus laser, and LIH groups survived less than 17, 19, and 20 days, respectively. The results of tumor weight are shown in Figure 6(D) and the tumors in LIH plus laser group nearly disappeared, the tumor weight of the saline, LI plus laser, and LIH groups were 1.24 ± 0.11, 1.08 ± 0.38, and 2.31 ± 0.29 g, respectively. The photos of tumors from different groups are shown in Figure 6(E).

Furthermore, the main organs (heart, liver, spleen, lung, and kidney) and tumors were also collected for histology analysis in order to evaluate the chronic toxicity of LI or LIH after the photodynamic therapy. It was found that these organs did not present an obvious abnormal morphology after the drug administration for 21 days, suggesting that LIH plus NIR irradiation at 1.5 W/cm^2^ for 3 min induced no toxicity to main organs (Supplementary Figure S4), but causethe destruction of tumor compact structure (Figure 6(F)). An obvious enlargement of spleen was observed in mice treated with saline, combined with the abnormal histopathology morphology of spleen in saline treated group indicated the immunological stress reaction of CT-26 tumor bearing mice, but no obvious damage of other organs in four groups were observed (Supplementary Figure S4). Tumor proliferation and apoptosis analysis are showed in Figure 6(G,H), it is obviously that the tumor treated with LIH plus laser was accompanied by high TUNEL positive but low Ki67 positive staining, which indicated the LIH plus laser promote apoptosis and inhibit proliferation. These give further evidence for efficient PDT mediated by LIH *in vivo*.

## Discussion

PDT is a clinical modality that employs a photosensitizer, an appropriate excitation light, and oxygen to generate cytotoxic ROS for the treatment of many localized and superficial cancers. However, the efficiency of PDT is badly hampered by tumor hypoxia (Dewhirst et al., 2000; Zhu et al., 2016; Feng et al., 2017) and the occurrence of hypoxia further induced worse treatment outcomes and tumor recurrence in PDT (Henderson & Fingar, 1987; Henderson & Dougherty, 1992; Maas et al., 2012). Over the past decade, various strategies have been explored for enhancing the ^1^O_2_ generation efficiency of photosensitizers and controlling their photodynamic action to improve the PDT efficacy. For example, dividing irradiation into light-dark circles has been investigated for better tumor reoxygenation by the blood (Curnow et al., 1999), however, this only affects PDT-induced oxygen depletion, whereas the preexisting hypoxia cannot be improved; moreover, the oxygen consumption during PDT always leading to an increased insufficient oxygen level in tumors, which can adversely affect the PDT efficiency in turn (Busch et al., 2010). Hyperbaric oxygen inhalation has also been used to increase tumor oxygen (Jirsa et al., 1991; Tomaselli et al., 2001; Maier et al., 2000b). However, vascular damage during PDT still prevents further oxygenation from hyperbaric blood (Fingar et al., 1988); besides, the potential toxic effects of excessive oxygen is still an impediment to its clinical use (Dong et al., 1987). To our knowledge, no existing techniques can effectively reverse the tumor oxygen content during PDT and achieve excellent therapeutic effect. Therefore, optimizing the efficacy with limited oxygen is of great importance for effective photodynamic therapy.

To address this challenge, herein we promote a liposome-based nanomedicine, LIH, for overcoming hypoxia and biological barriers, which could synchronously deliver oxygen and photosensitizer to tumors for alleviation of hypoxia tumor microenvironment and enhanced PDT. The Hb encapsulated in LIH plays the role of delivering oxygen to tumor, improved the tumor hypoxia microenvironment, and provided oxygen for the generation of ROS which enhanced the efficiency of PDT. LIH exhibited better oxygen combination ability for the protection of liposomes (Figure 1(B)). We found that LIH could synchronously deliver oxygen and photosensitizer into cells incubated in hypoxic environment (Figure 2(A-C)) and the alleviation of cellular hypoxia was successful (Figure 2(D-G)). Compared with the previously methods such as oxygen inhalation, our LIH liposomes could effectively get into the inside of cells and deliver oxygen molecules into cells, thus the hypoxia state of cells would be greatly improved.

Interestingly, the *in vivo* biodistribution experiments also demonstrated that the LIH could effectively be accumulated in the subcutaneous and orthotopic CT-26 tumors (Figure 4). This might be attributed to the similarity to RBC that exhibit excellent biological barriers overcoming abilities that are able to delay uptake by the mononuclear phagocyte system, preferential binding to the endothelium, and decrease retention in reticuloendothelial system organs. We also investigated the alleviation of tumor hypoxia after intravenous injection of LIH into mice bearing tumors by immunohistochemical staining (Figure 5(A-I)) and monitored the oxygen level using *T*_2_-weighted magnetic resonance imaging (Figure 5(J-K)). The improved state of tumor hypoxia could be maintained for about two days (Figure 5(G-I)) and this time span is not too long. Nevertheless, it is very important in PDT to arrange the time interval of administration and the optimized time point of adding laser irradiation to tumors to obtain an excellent therapeutic effect.

Our results indeed demonstrated that the sufficient oxygen provided by LIH would not only improve the hypoxia of tumor but also enhance the efficacy of PDT. We verified that due to the lack of oxygen, the generation of ROS which play a major role in killing the tumor cells was impeded (Figures 1(F-G) and 3(A,B)). Correspondingly, the generation of ROS and cytotoxicity of cells incubated with LIH showed that the PDT effect was dramatically enhanced (Figure 3(A-D)). Eventually, we proved that the LIH enhance the efficiency of PDT on tumor bearing mice, inhibit the growth of tumor via its advantage of oxygen-carrying (Figure 6), also was seen to induce the highest PDT efficiency in tumors (Figure 6(A)) because these liposomes carried enough oxygen, the hypoxia condition of the tumor was improved, and more ROS was seen to be produced by ICG (Figure 5). ROS trigger oxidative damage of tumors and induce complete suppression of tumor growth and 100% survival rate of mice, which were also in good health condition. These excellent effects were further confirmed by Ki67 staining for cell proliferation and TUNEL staining for apoptosis (Figure 6(G-H)) in tumor tissues. Moreover, as hypoxia is effectively improved, this strategy could be applied to other therapies in which oxygen is a critical component, such as radiotherapywhich is a widely used first-line treatment for many cancer types (Song et al., 2016).

## Conclusions

In this work, we reported a synchronous oxygen and photosensitizer delivery LIH liposome for dramatically enhancing PDT against hypoxic tumors by sufficient supply of oxygen. It’s alleviation of hypoxia tumor microenvironment and enhanced photodynamic therapy were also systematically investigated. Our data indicated that the LIH exhibited outstanding ability as an oxygen carrier and could generate massive ROS under the laser irradiation in hypoxia condition, which suggested the possibility for further *in vivo* research. Moreover, the LIH could be effectively internalized by cells incubated in hypoxia environment and down-regulated hypoxia-associated proteins of cells. Thus, sufficient ROS could be produced to kill the cells efficiently under the NIR laser irradiation. The *in vivo* results indicate that LIH could accumulate into subcutaneous and deep orthotopic tumors, which indicated that large amount of oxygen could be delivered to tumor for ICG to generate toxic ROS under NIR laser irradiation and this was further identified by a *T*_2_-weighted MRI. Furthermore, the expression level of HIF-1α and VEGF in LIH treated tumors was obvious down-regulated and this state was seen to be maintained for several days. Thus, the improvement of tumor hypoxia environment and dramatically enhanced PDT efficacy against hypoxia tumor *in vivo* was true . This work significantly demonstrated that LIH may be a unique type of safe nanoplatform promising for cancer PDT, particularly for enhancing cancer treatment outcomes via modulating the unfavorable tumor microenvironment.

## Supplementary Material

IDRD_You_et_al_Supplement_Content.pdf
