# Supplementary material for: Synchronous delivery of oxygen and photosensitizer for alleviation of hypoxia tumor microenvironment and dramatically enhanced photodynamic therapy
Source: Drug Deliv. 2018 Feb 20;25(1):585–99. doi: 10.1080/10717544.2018.1435751 (PMC6058564; doi:10.1080/10717544.2018.1435751)
Supplement: IDRD_You_et_al_Supplement_Content.pdf [file IDRD_A_1435751_SM3342.pdf]

Supplementary data

Synchronous delivery of oxygen and photosensitizer for alleviation of hypoxia tumor microenvironment and dramatically enhanced photodynamic therapy

Xiaomeng Guo[1,#], Jiaxin Qu[1,2,#], Chunqi Zhu[1], Wei Li[1], Lihua Luo[1], Jie Yang[1], Xiaoyi Yin[2], Qingpo Li[1], Yongzhong Du[1], Dawei Chen[2], Jian You[1]*

Authors' Affiliations:

[1]College of Pharmaceutical Sciences, Zhejiang University, 866 Yuhangtang Road, Hangzhou, Zhejiang 310058, P. R. China

[2]Department of Pharmaceutics, School of Pharmaceutical Science, Shenyang Pharmaceutical University, 103 Wenhua Road, Shenyang 110016, P. R. China

[#]These authors contributed equally to this work.

* Corresponding Author:

Jian You, College of Pharmaceutical Sciences, Zhejiang University, 866 Yuhangtang Road, Hangzhou 310058, Zhejiang, China. Office: 086-571-88981651 Fax: 086-571-88208439Email: youjiandoc@zju.edu.cn.

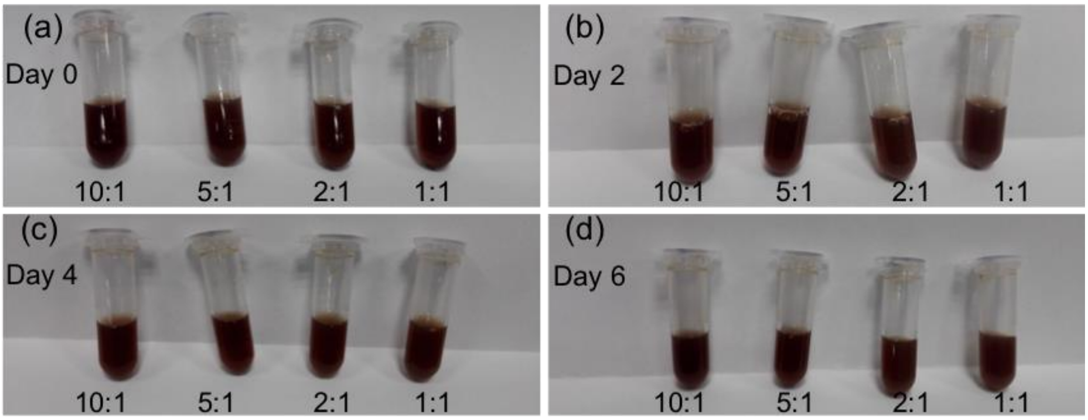

Figure S1. The stability of LIH liposomes prepared with different ratios of phosphatide at day 0, 2, 4 and 6.

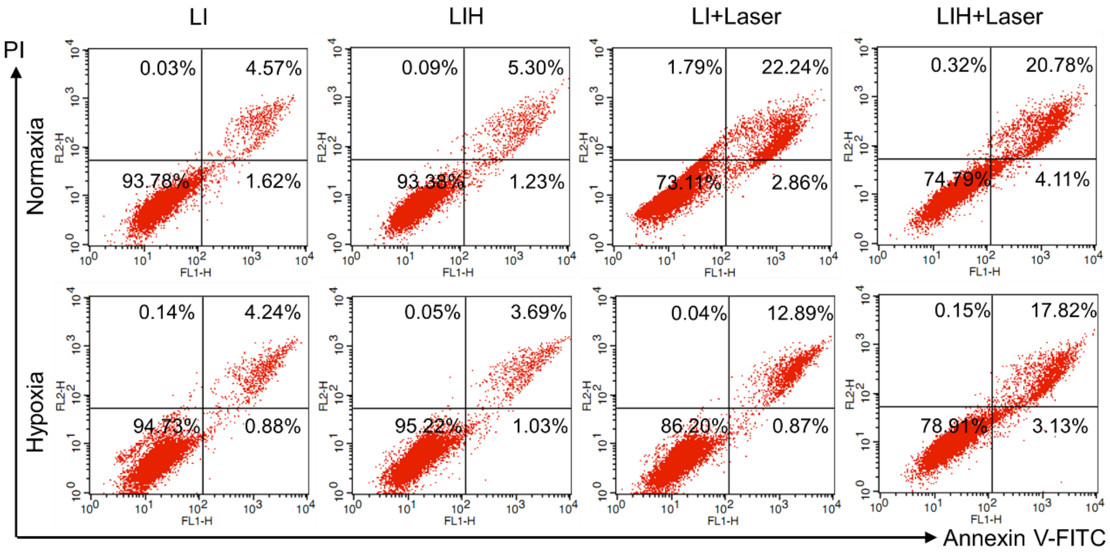

Figure S2. Apoptotic results of LI and LIH against CT-26 cells in normaxia and hypoxia environment without or with laser irradiation (808 nm, 1 W/cm^2, 1 min), the concentration of ICG was 5 ug/mL.

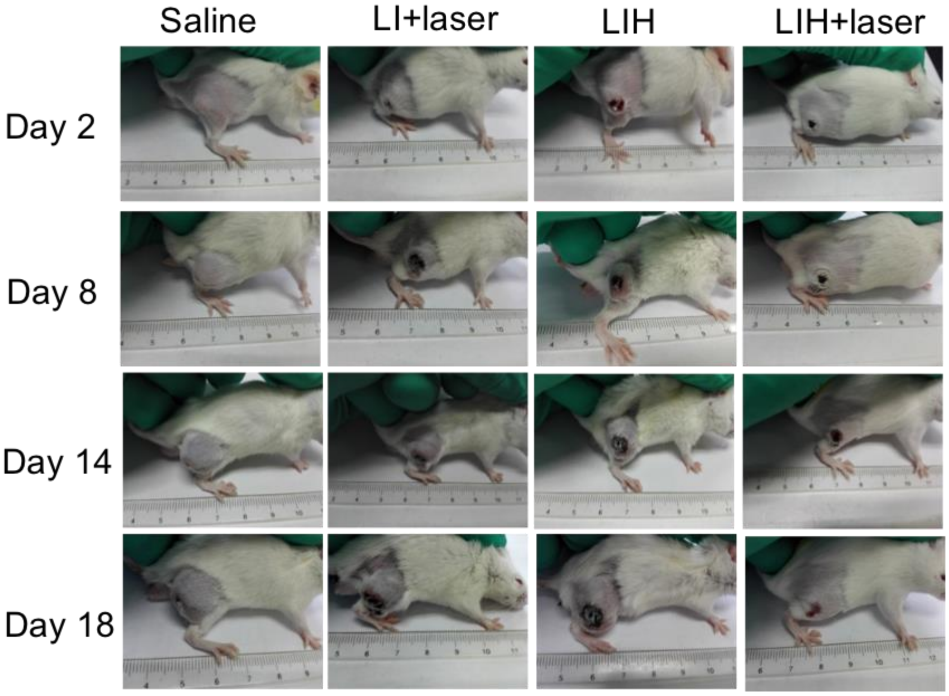

Figure S3. Representative images of mice during the enhanced PDT treatment.

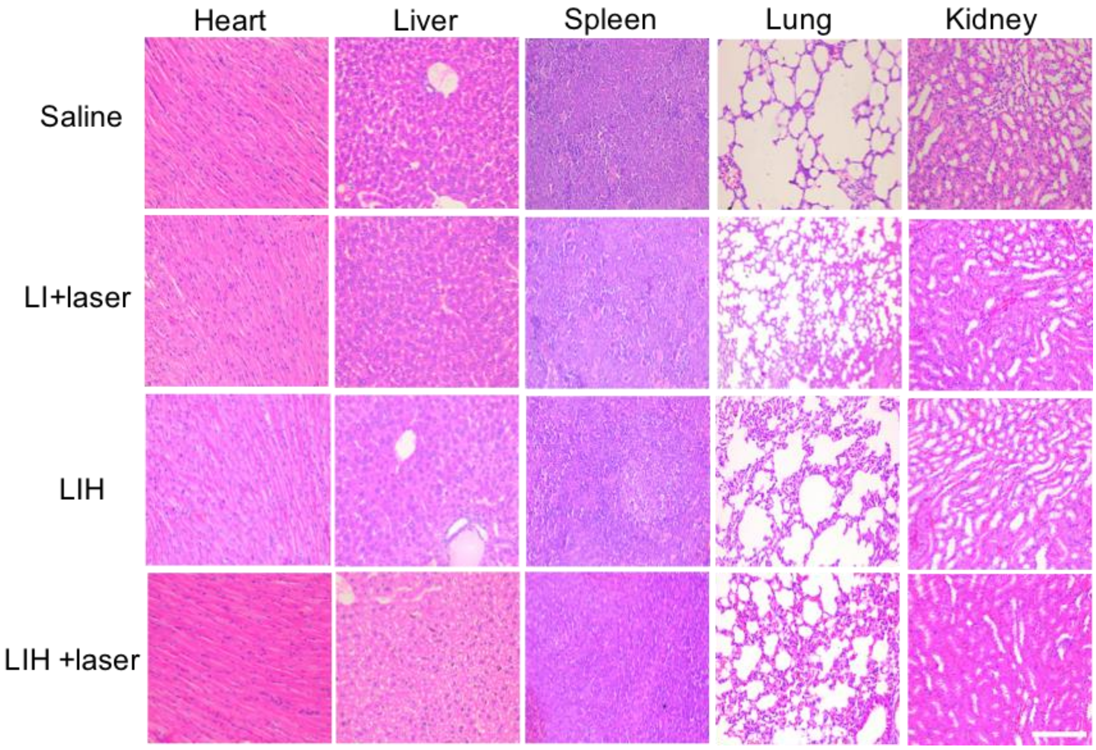

Figure S4. Systemic toxicity evaluation via hematoxylin and eosin (H&E) staining of main organs (heart, liver, spleen, lung and kidney) after experiments. Scale bars, 50 μm.
